# Evaluating Joint Angle Data for Clinical Assessment Using Multidimensional Inverse Kinematics with Average Segment Morphometry

**DOI:** 10.1101/2024.09.03.611088

**Published:** 2024-09-07

**Authors:** Rachel I Taitano, Valeriya Gritsenko

**Affiliations:** Department of Neuroscience, School of Medicine, West Virginia University, Rockefeller Neuroscience Institute, West Virginia University, Morgantown, USA; Department of Human Performance, Division of Physical Therapy, School of Medicine, West Virginia University, Department of Neuroscience, School of Medicine, West Virginia University, Rockefeller Neuroscience Institute, West Virginia University, Morgantown, USA

**Keywords:** Movement analysis, Motor deficits, Disabilities, Motion capture technology, Upper extremity movements, Virtual reality, Dynamic inertial model, Model scaling, Rehabilitation, Remote healthcare, Quantitative data, Movement redundancy

## Abstract

Movement analysis is a critical tool in understanding and addressing various disabilities associated with movement deficits. By analyzing movement patterns, healthcare professionals can identify the root causes of these alterations, which is essential for preventing, diagnosing, and rehabilitating a broad spectrum of medical conditions, disabilities, and injuries. With the advent of affordable motion capture technologies, quantitative data on patient movement is more accessible to clinicians, enhancing the quality of care. Nonetheless, it is crucial that these technologies undergo rigorous validation to ensure their accuracy in collecting and monitoring patient movements, particularly for remote healthcare services where direct patient observation is not possible. In this study, motion capture technology was used to track upper extremity movements during a reaching task presented in virtual reality. Kinematic data was then calculated for each participant using a scaled dynamic inertial model. The goal was to evaluate the accuracy of joint angle calculations using inverse kinematics from motion capture relative to the typical movement redundancy. Shoulder, elbow, radioulnar, and wrist joint angles were calculated with models scaled using either direct measurements of each individual’s arm segment lengths or those lengths were calculated from individual height using published average proportions. The errors in joint angle trajectories calculated using the two methods of model scaling were compared to the inter-trial variability of those trajectories. The variance of this error was primarily within the normal range of variability between repetitions of the same movements. This suggests that arm joint angles can be inferred with good enough accuracy from motion capture data and individual height to be useful for the clinical assessment of motor deficits.

## Introduction

In the United States (US), 1 in 4 adults (61 million) live with a disability [1]. The majority of disabilities are often linked to deficits in movement, which can be effectively evaluated through movement analysis. Movement analysis seeks to understand the cause of altered movement patterns, assisting with the prevention, identification, and rehabilitation of a wide array of diseases, disabilities, and injuries [2–9]. Moreover, early identification plays a major role in combating disease progression, facilitating interventions using precise measurements of small changes in movement characteristics [10–13]. Movement analysis commonly relies on practitioner observations and visually assessed rating scales [14–19]. More recently, metrics based on inverse kinematics, which measures joint angles from tracked body segments, are gaining widespread adoption as they help to reduce inter-rater variability and standardize movement analysis [20]. Joint angles show improvement in arm function after rehabilitation of stroke survivors with higher inter-rater reliability than clinical tests [21–23]. Our team has also demonstrated the feasibility of inverse-kinematics-based assessment of complex arm impairment after stroke and of measuring shoulder range of motion (ROM) deficits after chest surgery [24–26]. Moreover, analysis of natural and unrestrained functional tasks with simultaneous movement of multiple joints is deemed crucial for post-stroke clinical assessment of changes in intersegmental coordination [27,28]. However, a significant challenge to the widespread adoption of motion-capture-based assessment is the uncertainty regarding how the accuracy of inverse kinematics algorithms impacts clinical metrics. In this study, we examined one aspect of this issue: how individual variability in arm segment lengths affects joint angle calculations relative to the normal ROM and the natural variability of multijointed movements.

Movement is a complex interplay between an individual’s neural control, body biomechanics, and interactions with the external environment. Because of our complex multijointed limbs with multiple degrees of freedom (DOFs), the same movement can be performed with different combinations of joint angles and muscle forces. This individual redundancy underlying inter-subject variability is clinically unaccounted for. Furthermore, the prevalence of movement disorders increases with age [29,30], thus sarcopenia and age-related neurological changes further confound individual differences [31–36]. To minimize the false-positive rate, the clinical tests typically used for movement analysis employ a low-resolution scoring system based on subjective observations by practitioners. This ensures only the most obvious movement deficits are detected, but this reduces the responsiveness and predictive validity of clinical tests and introduces ceiling effects in patients with mild motor deficits [18,37]. This poses the challenge of using movement analysis to distinguish the typical variability due to this redundancy from the abnormal changes in movement caused by damage or movement disorder. Here we investigate the extent to which redundancy affects joint angle measurements.

**Figure 1.**
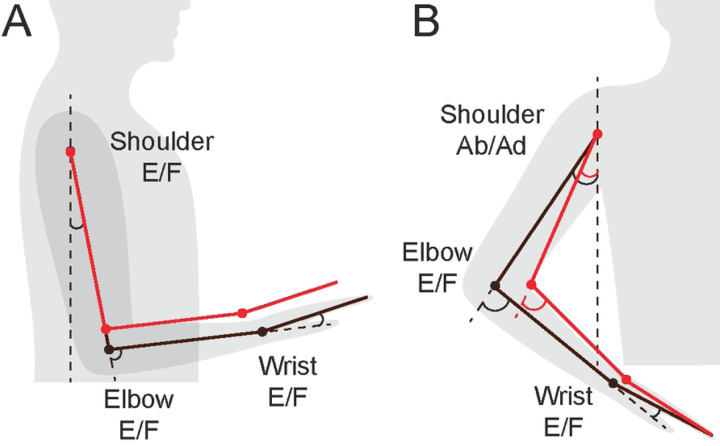
Illustration of the effect of 10% change in segment lengths on the resulting arm pose. Red segments are 10% shorter each than the black segments. Dashed lines show axes against the joint angles are measured. E/F stands for extension/flexion degree of freedom of the corresponding joint. **A.** When the joint angles are kept the same, the changes in limb segment lengths change the location where the hand can reach. **B.** When the tip of the hand is kept the same, the changes in limb segment lengths change joint angles.

The lengths of the segments between each joint are the anthropometric measurements that are often used to customize computer models when processing motion capture data [38]. The individual morphometry of body segments has been documented in several ergonomic studies last century. They employed different cohorts of primarily young adults, mostly male. For example, one of these studies recruited 39 male fighter pilots into three cohorts divided based on volumetric body types (thin, muscular, and rotund) [39]. Based on this and other classical studies the mean segment length proportions to height were summarized by Winters and others [40–42]; these average proportions are widely used to scale biomechanical models. A recent study of 445 male and 401 female sportspersons has largely found similar proportions [43]. However, there is individual variability in the segment lengths and their proportions to the overall body height. The segment lengths are crucial for joint angle calculations as they define the kinematic chain of the limb. For example, altering the length of arm segments by 10% would place the hand at a different location relative to the trunk given the same joint angles (Fig. 1A). Conversely, when reaching toward the same location in space, the joint angles would be different for different segment lengths (Fig. 1B). Yet, it is not always feasible to measure individual segment lengths precisely particularly during remote assessment. Therefore, understanding the influence of individual variability in segment lengths on joint angle calculations is crucial for defining the accuracy of assessment algorithms aimed at measuring movement deficits. Here, we compared the accuracy of joint angle calculations using arm segment lengths derived from average body proportions to those obtained from direct measurements, relative to the typical variability in joint angles observed in repeated reaching movements.

## Methods

### Participants and experimental design

Institutional Review Board of West Virginia University approved the experimental protocol (Protocol #1311129283). Volunteers were recruited through fliers distributed around Morgantown, WV. The data collection started on March 12^th^, 2014, and ended on July 25^th^, 2016. Volunteers provided their written informed consent prior to the start of the experiment. The signing of the informed consent form was witnessed by a second member of the research team.

Nine healthy human volunteers (22.8 +/− 0.67 years, 6M:3F) performed a center-out reaching task comprising reaching toward visual targets presented in virtual reality as described in detail in Olesh et al. [44]. Target placement within the virtual task was adjusted based on each participant’s arm length to minimize inter-subject variability in starting joint angles (Fig. 1A). Arm motion was recorded at 480 Hz temporal resolution using active markers coordinates of which were resolved at submillimeter spatial resolution by the Impulse system (PhaseSpace). The markers were placed on bony landmarks according to standard practices [45]. Additionally, the individual self-reported height was recorded and the lengths of the humerus, radial/ulnar, and hand segments were measured at the time of the experiment. The length of the humerus was measured between the acromion and the lateral epicondyle. The length of the radius/ulnar segment was measured between the lateral epicondyle and the location between the styloid processes of the ulna and radius. The length of the hand segment was measured between the median of the styloid processes and the tip of the index finger. The index finger was used because the participants were instructed to point with that finger and its tip was instrumented with the marker.

### Data analysis

A dynamic model of the arm and hand with 27 degrees of freedom was developed using Simscape Multibody toolbox MATLAB [46]. Here, we compared two methods for scaling the model segments. The first method, termed here the Average method, relied on published anthropomorphic dimensions as fractions of individual height [40,47]. The second method, termed here the Individual method, used the measured segment lengths, while the rest of the dimensions were kept the same as in the first method. Next, joint angles were calculated for all degrees of freedom at the shoulder, elbow, radiusulnar, and wrist joints as described in detail in Bahdasariants et al. [46] using models scaled in two ways. Briefly, averaged trajectories of marker coordinates were used to drive the movement of virtual markers attached to model segments through linear springs. The accompanying angular trajectories of three shoulder degrees of freedom (DOFs for flexion/extension, abduction/adduction, and internal/external rotation), one elbow DOF (flexion/extension), one radioulnar DOF (pronation/supination), and two wrist DOFs (flexion/extension and radial/ulnar deviation) were recorded for each reaching movement (Fig. 1).

**Figure 2.**
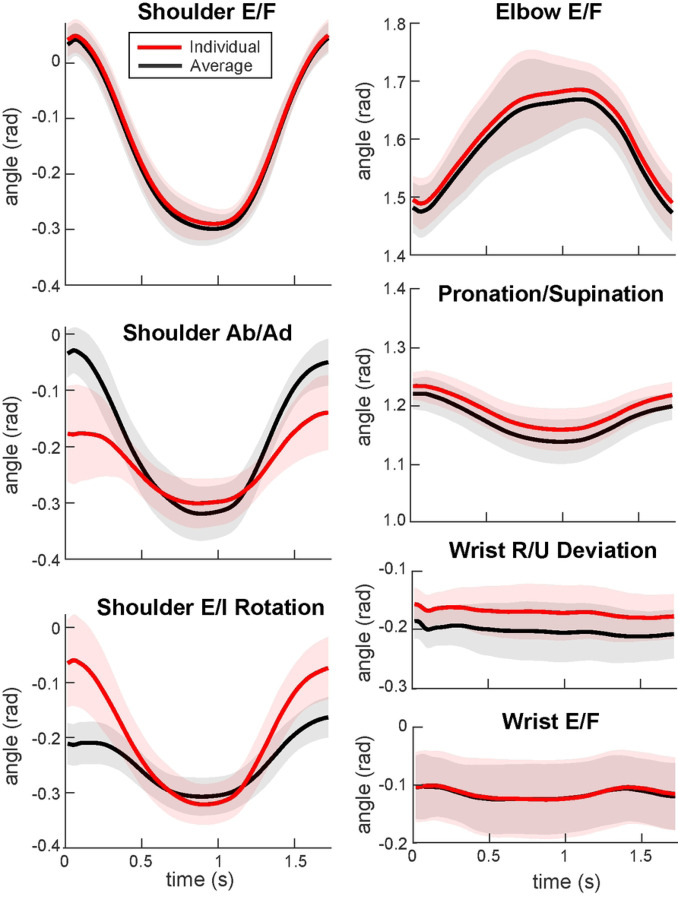
Example of the differences between joint angle trajectories calculated with models scaled in two ways for a single movement in one direction (and back) performed by one participant. Joint angle trajectories were calculated from musculoskeletal models scaled using average proportions (black) and individual arm segment lengths (red). Thick lines show average trajectories and shaded areas show standard deviation across 15 repetitions for the same movement. Abbreviations: E/F = extension/flexion; Ab/Ad = abduction/adduction; E/I = external/internal; R/U = radial/ulnar.

Statistical analysis compared the inter-trial variability to the changes in joint angles caused by the two methods of segment length calculations. The inter-trial variability for a given movement direction was measured as the standard deviation (SD) of the angular trajectory of each DOF from simulations with average segments (Fig. 2, grey shaded areas). SD values were averaged over the whole duration of each movement toward a target per DOF per subject. The effect of segment length variability on joint angles was measured as the root mean squared error (RMSE) calculated between the angular trajectories derived by models scaled with both methods for each corresponding reaching movement per subject (Fig. 2). The units of both measures were degrees. Statistical analysis was performed on the difference between SD and RMSE values in degrees using repeated-measures analysis of variance (fitrm function in MATLAB). The subject’s sex assigned at birth served as a between-subject factor, the within-subject factors were movement direction (Target # 1:14, Fig. 3 insert) and DOF (5 DOFs of major shoulder, elbow, and forearm joints).

## Results

The segment lengths measured in our small-cohort study were representative of the previously reported data for taller individuals (Table 1). The segment lengths estimated with the Average method were less variable than those measured directly (Individual method). This is likely due to the lower error in measuring the height vs. segment lengths. Additionally, more rigorous methods were utilized by the earlier studies that measured segment length that supported the derivation of the proportions used by the Average method [41]. The variability in the measurement of individual segment lengths fell within the natural variability between individuals, thus it is unlikely to limit the validity of the study conclusions.{Citation}

Joint angle trajectories calculated using models scaled with the Average and Individual methods were very similar (Fig. 2). For some DOFs, the initial and starting angles were not the same (Fig. 2, shoulder abduction/adduction and rotation DOFs). This is because different joint angles are needed to reach the same target position with different segment lengths as illustrated in Fig. 1B. The difference in the initial joint angles for the example in Fig. 2 was about 0.1 radians or 6 degrees, accounting for about 3% and 7% of the total range of motion (ROM) for shoulder abduction/adduction and external/internal rotation respectively (Table 2). Repeated-measures ANOVA analyzing the differences between SD and RMSE values had no significant effects (Table 3) indicating that the errors caused by different segment length calculations largely fell within normal inter-trial variability.

**Figure 3.**
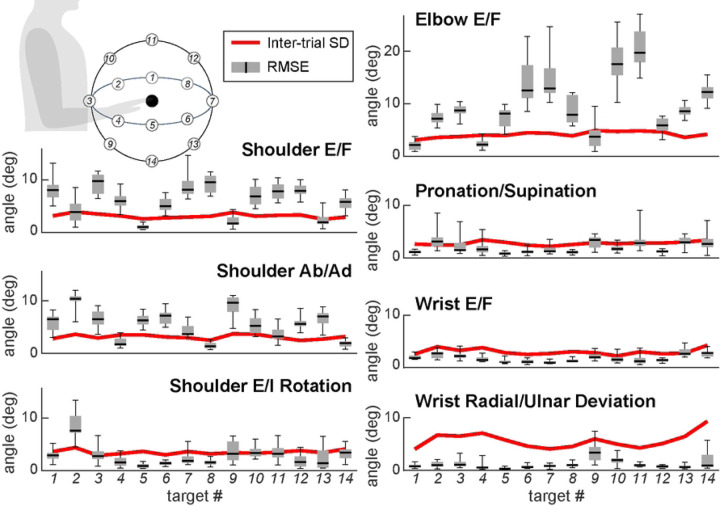
Root mean squared error (RMSE) between joint angles calculated using the two scaling methods. RMSE was averaged across all subjects for each target location and degree of freedom. Thick black lines show median RMSE values, shaded areas show 2dn and 3rd quartile ranges, whiskers show maximal ranges of RMSE values. Red lines show the average inter-trial standard deviation for corresponding movements and degrees of freedom. The insert shows the locations of targets 1–14 relative to the subject’s position (drawn not to scale). Abbreviations: E/F = extension/flexion; Ab/Ad = abduction/adduction; E/I = external/internal.

Join DOFs are coupled by the kinematic chain of the limb, therefore for movements in different directions, different combinations of joint angles are affected by the differences in segment lengths. Altering segment lengths had the largest effect across all reaching directions and participants on shoulder flexion/extension, shoulder abduction/adduction, and elbow flexion/extension angles (Fig. 3). The angles for the rest of the DOFs largely fell within the natural variability between repetitions for the same movement as indicated by the statistical analysis (Fig. 3, red lines). The largest RMSE between the angles calculated with the two types of segment scaling were 10 degrees (6% of ROM) for shoulder flexion/extension during movement toward target 3, 11 degrees (5% of ROM) for shoulder abduction/adduction during movement toward target 2, 8 degrees (9% of ROM) for shoulder external/internal rotation during movement toward target 2, 20 degrees (15% of ROM) for elbow flexion/extension during movement toward target 11, 4 degrees (3% of ROM) for pronation/supination during movement toward target 9, 3 degrees (2% of ROM) for wrist flexion/extension during movement toward target 13, 3 degrees (6% of ROM) for wrist radial/ulnar deviation during movement toward target 9 (Table 2). The slight variations in joint angles, which make up a minor part of the healthy ROM, are likely too subtle to be identified during professional evaluations of movement deficits. If we apply the same criteria to algorithms, it implies that both methods of segment length measurement could be suitable for evaluating movement deficits.

## Discussion

Here we have quantified joint angle trajectories using two methods, individually measured segment lengths and inferred segment lengths based on individual height and average body proportions. The Average method of calculating the segment lengths altered the values of shoulder and elbow joint angles the most. The differences in angles ranged from 0 to 20 degrees or from < 2 to 15 % of the total range of motion of the corresponding DOF. Most of these differences fell within the normal variability in joint angles that occurs across repetitions of the same movements by an individual. Both visualizations of the same movement during our dynamic inverse kinematic simulations using models with different segment lengths appeared normal. Therefore, the largest discrepancies between the joint angle values calculated using the two methods observed here are unlikely to confound the evaluation of movement quality. Instead, the assessment of patient quality of movement is mostly sensitive to the shape of joint angle trajectories [24,26], which were not affected by the different segment measures (Fig. 2). Moreover, the ROM assessment is even less sensitive to segment length estimates as it relies on the observation of isolated single-joint movements with limited kinematic redundancy. Our team has shown in an earlier study that it is feasible to measure the active and passive range of shoulder motion using noisy camera-based motion capture [25]. This suggests that arm joint angles can be inferred with *good enough* accuracy from motion capture data and individual height to be useful for the clinical assessment of movement quality and ROM.

Movement abnormalities or dysfunctions are commonly evaluated in medical practice using systematic observation and analysis of patient’s movements by trained medical professionals. To ensure that systematic observation is reliable, valid, and quantifiable, the professional observation of standardized sets of movements is often mapped onto clinical scales. These can be basic 3-point scales, where 1 indicates no movement, 2 indicates limited ROM or abnormal movement quality, and 3 indicates normal movement as in the Fugl-Meyer Assessment [17]. These can also be a more dexterous 5-point scale with more nuanced descriptions of the effort required to make the movement as in the Wolf Motor Function Test [48,49]. These scales provide a much lower resolution metric of the motion compared to the resolution offered by even the lowest-quality motion capture [24,50–52]. Therefore, together with our results, there is compelling evidence for the replacement of subjective clinical scales with objective metrics based on joint kinematics obtained from motion capture.

Another component of systematic observation is the detection of compensation strategies [53]. For example, a stroke patient might use their trunk muscles more to compensate for weakened arm muscles during a reaching task. Motion capture-based assessment can detect this deviation from the normal pattern of joint ROM as the reduced active ROM at the shoulder and increased active ROM of the trunk. These joint angle measures can be converted into a clinically relevant scale of relative deviations from the neurotypical motion pattern. Unlike subjective assessments prone to human error, these metrics will not have inter-rater variability. Objective metrics obtained from motion capture will be as sensitive as the quality of motion capture and normative data allows [24,26,52,54–57]. This objective data will also enable a better understanding of a patient’s unique compensatory strategies and help therapists to tailor rehabilitation programs more effectively.

## Figures and Tables

**Table 1.** Segment lengths calculated with the two methods and data from two reference studies.

Length measurements	From Dempster et al. 1955 (cm)	From Canda 2009 (cm)	Individual method (cm)	Average method (cm)	Difference (cm)
Humerus	35.3 ± 1.7	M: 33.9 ± 2.1 F: 31.3 ± 1.9	35 ± 2.5	34 ± 1.2	2.0 ± 1.9
Radius/ulnar	27.3 ± 1.1	M: 26.2 ± 1.7 F: 23.7 ± 1.5	26 ± 1.7	26 ± 0.9	1.3 ± 0.8
Hand	19.1 ± 1.0	M: 19.6 ± 1.1 F: 17.9 ± 0.9	21 ± 1.8	19 ± 0.7	1.8 ± 1.5
Height	175.1 ± 4.3	M: 179.4 ± 9.1 F: 166.5 ± 7.6	180 ± 6.4		

Values are means ± standard deviation.

**Table 2.** Angle errors between scaling methods relative to the total joint range of motion

Joint DOF	ROM (deg)	RMSE across all target locations	RMSE / ROM
Shoulder abduction/adduction	220	2.3065	0.0104
Shoulder flexion/extension	180	6.4383	0.0358
Shoulder internal/external rotation	90	5.7618	0.0640
Elbow flexion/extension	130	5.9418	0.0457
Supination/pronation	160	6.1975	0.0387
Wrist flexion/extension	150	2.6294	0.0175
Wrist radial/ulnar deviation	50	2.2168	0.0443

ROM stands for range of motion; RMSE stands for root mean squared error; DOF stands for degree of freedom.

**Table 3.** Repeated-measures ANOVA results.

	Sum of Squares	DF	F-statistic	*p-value*
Intercept	269.26	1	1.81	0.25
Gender	308.05	1	2.07	0.22
Intercept:Target	256.86	1	2.60	0.18
Gender:Target	181.73	1	1.84	0.25
Intercept:DOF	498.88	1	5.41	0.08
Intercept:Target:DOF	342.13	1	4.16	0.11
Gender:Target:DOF	169.39	1	2.06	0.22

Intercept refers to the baseline level of the dependent variable when all factors are set to zero; a semicolon shows interaction effects between factors; DF is degrees of freedom.

## Data Availability

The data used for statistical analysis, such as RMSE and SD per subject, per DOF, and per movement, are shared online (https://doi.org/10.6084/m9.figshare.26885002.v1).
